# A Comprehensive Review of Radiomics in Pulmonary Nodule Management: Clinical Applications and Standardization Dilemmas

**DOI:** 10.2174/0115734056460566260609044755

**Published:** 2026-06-22

**Authors:** Guo Chen, Zuxin Wang, Hua Jiang

**Affiliations:** 1 Department of Radiology, Kunming Medical University, Kunming 650500, People’s Republic of China;; 2 Interventional Radiology Group, Multidisciplinary Tumor Treatment Center, Department of Oncology, Kunming Medical University Affiliated Second Hospital, Kunming, 650101, People’s Republic of China

**Keywords:** Pulmonary nodules, Lung cancer, Radiomics, Precision medicine, Radiogenomics, Fatal malignant tumour

## Abstract

Lung cancer is the most common and fatal malignant tumour. Early detection and treatment are likely to reduce mortality, but most pulmonary nodules identified during routine health checks are harmless. Consequently, a clear distinction between benign and malignant nodules is vital to improve early detection and reduce unnecessary interventions. Radiomics, a new omics technology, can be used to extract high-dimensional quantitative features from medical images, providing a profound understanding of tumour pathophysiology. Radiomics has attracted the attention of medical researchers since its formal definition by the Dutch researcher Lambin *et al*. in 2012. The number of research papers on radiomics has grown tremendously over the past few years. At present, it is used to predict pulmonary nodule malignancy, for noninvasive risk stratification, for integration with genomics to identify genetic mutations associated with lung cancer, and for evaluation of therapeutic responses. With this review, we summarise the literature on radiomics of pulmonary nodules, discuss how it could be used in nodule management, and address the current challenges and future directions for improving precision oncology.

## 1 INTRODUCTION

Lung cancer is the most prevalent malignancy and the leading cause of cancer-related mortality in China. According to statistics, there were about 1,060,600 new lung cancer patients in China in 2022, which accounted for 22.0% of all malignant tumours. It was reported that 733,300 patients died of lung cancer, which is 28.5% of all cancer deaths [1]. The overall 5-year survival rate for lung cancer in China is 19.7%. However, for patients diagnosed with stage I, the 5-year survival rate is 77%–92% [2]. These statistics highlight the necessity to detect and intervene early to achieve better survival outcomes.

Lung cancer in its early stages is largely asymptomatic, with symptomatic patients usually being diagnosed at advanced stages. As such, regular check-ups play a very important role in early detection. Routine check-ups conducted using high-resolution computed tomography (HRCT) have led to the diagnosis of a large number of pulmonary nodules. Nevertheless, very few of these nodules develop into malignancy. Pulmonary nodule aetiology, imaging, and growth patterns are extremely heterogeneous, creating a significant clinical dilemma regarding the choice between active surveillance and surgical intervention and increasing the risk of overtreatment [3]. Therefore, accurate preoperative differentiation between benign and malignant nodules is essential for optimizing treatment strategies and improving patient prognosis.

In 2012, Lambin *et al*. [4] coined the term radiomics to refer to the process of extracting a large number of quantitative features of medical images and their analysis using computational methods to find clinically significant biomarkers. Radiomics has since revolutionized diagnostic imaging and is now a pillar in precision medicine. New developments in pulmonary radiomics have directed attention to the prediction of malignancy, which allows quantification of subvisual characteristics, such as volumetric pattern, textural heterogeneity, surface abnormalities, and parenchymal changes, which are not assessable through traditional radiologic methods using manually assessed parameters like size, shape, and density [5]. Despite its relatively recent emergence, the accumulated evidence indicates the potential of radiomics to transform the process of enhancing the classification of lung nodules, the risk of malignancy, individualized treatment development, and prognostic interpretation.

This review will serve to narrate the workflow of radiomics, general recent developments in the application of radiomics to the precise diagnosis and treatment of pulmonary nodules, and critically assess the current issues and future avenues of research in this emerging field.

### 1.1 The Origin and Evolution of Radiomics

In 2012, Lambin *et al*. [4] developed the term “radiomics,” which is based on the recognition that medical imaging contains a wealth of pathophysiological information that can be fully exploited by advanced image processing techniques. Its clinical use was highlighted in their 2017 paper [6]. In particular, they showed that radiomic signatures together with clinical data, genomic profiling, and immunohistochemical biomarkers could be combined to enhance early tumour detection, accuracy of grading, precision therapy, and prediction of outcomes [7, 8]. Radiomics has since become one of the building blocks of precision medicine.

The rapid advancement of this field can be attributed to several key developments: (1) progress in medical imaging technology, as modern modalities such as CT, MRI, and PET-CT offer superior spatial and contrast resolution, enabling high-quality feature extraction; (2) the emergence of machine learning approaches, particularly deep learning, which have revolutionised automated feature quantification and pattern recognition; and (3) enhanced computational capacity, allowing for efficient processing of large imaging datasets and the development of robust radiomic models.

### 1.2 Workflow of Pulmonary Radiomics

The standard workflow of pulmonary radiomics is illustrated in Fig. (**1**) and typically involves the following steps:

#### 1.2.1 Image Acquisition and Data Collection

Image acquisitions in pulmonary radiomics typically involve chest CT or PET scans that capture pulmonary nodules or lung cancer lesions at the relevant anatomic levels. One of the major problems in this process is the fact that imaging protocols differ across institutions. Several factors may influence the consistency and quality of CT images, including the heterogeneity of CT scanners used, radiation dose, scanning parameters (*e.g*., pixel size, slice thickness), contrast agent usage, and patient-related factors (*e.g*., breath-hold consistency, body habitus). In addition, variation in the post-processing parameters following the reconstruction of CT images is also a source of data inhomogeneity [9].

To ensure reproducible and reliable feature extraction, the use of standardized acquisition parameters (slice thickness, tube voltage, tube current) is essential. Nevertheless, the absence of standardized imaging standards across the world continues to be a significant constraint in radiomics research. Addressing this limitation would significantly enhance the clinical applicability and robustness of radiomic models. In reality, high-resolution thin-slice imaging is typically preferred.

#### 1.2.2 Image Segmentation

Image segmentation refers to the process of delineating pulmonary nodules or lesions from the normal lung tissue on chest CT or PET scans. Region of interest (ROI) segmentation specifically refers to defining the boundaries of pathological regions. Solid nodules are defined fairly easily, but in subsolid or ground-glass nodules, segmentation is more difficult and disputed. Segmentation techniques can be manual, semi-automatic, or automatic. Manual segmentation is time-consuming and labor-intensive. Moreover, due to inter-operator variability, it often suffers from poor objectivity and reproducibility. Semi-automatic and automatic techniques offer improved efficiency and consistency, but they are not sufficiently accurate to segment nodules that are close to vital pulmonary structures (hilum, pleura, vasculature). Therefore, manual revision is typically needed.

In more recent times, machine learning and deep learning methods have been increasingly employed for automated nodule segmentation. Some studies extend the analysis to perinodular regions, necessitating segmentation of a larger area, whereas in other instances, full ROI annotation is not required [10].

(1) Feature Extraction, Statistical Analysis, Model Construction, and Evaluation:

After image segmentation and ROI delineation, feature extraction is achieved by commercial or open-source software. The extracted radiomic features can be broadly divided into three categories: morphological features (size, shape, volume), intensity-based features (lesion density and intensity distribution), and texture features (spatial structural patterns that show tissue heterogeneity) [11]. Then, statistical analyses are applied to identify clinically relevant features for incorporation into radiomic models, which are then interpreted for clinical application.

(2) Over the last few years, there has been increasing adoption of artificial intelligence (AI) and deep learning into radiomic processes in areas such as feature extraction, statistical analysis, and model building. Machine learning algorithms are increasingly employed to identify and classify features, thereby improving the accuracy of nodule segmentation as well as the diagnostic and prognostic performance. Deep learning, a subset of machine learning, has become particularly prominent. Specifically, convolutional neural networks (CNNs) have demonstrated greater effectiveness and precision in handling large-scale sets of features compared to traditional radiomic processes [12, 13].

(3) The training and validation datasets must be based on clinically verified cases (*e.g*., biopsy or postoperative histopathology) to guarantee the clinical quality and accuracy of radiomic models. Among the problems that are still relevant is model robustness across institutions, because there is inter-center variability in image acquisition, segmentation, feature extraction, and analysis techniques. These are the limitations that will be vital to address to improve the generalizability and clinical applicability of radiomic models.

### 1.3 Applications and Workflow Integration of Radiomics in Pulmonary Nodule and Lung Cancer Management

Radiomics enables clinicians to characterize pulmonary nodules accurately and noninvasively, predict tumor mutational burden (TMB) from imaging phenotypes in lung cancer, and evaluate treatment responses in malignant lesions. Such quantitative imaging features support clinical decision-making using imaging data alone, thereby minimizing the necessity of further invasive procedures (Fig. **2**). Incorporating radiomics throughout pulmonary nodule management could enhance cost-effectiveness and significantly improve patient outcomes.

### 1.4 Radiomics-Based Classification of Pulmonary Nodules

Once a CT scan identifies an indeterminate nodule in the lung, the next step is to determine its malignancy probability. The existing clinical models that categorize nodules according to clinical variables and the characteristics of images used as predictors are often inconsistent and have low accuracy [14-16], which can still be improved. As has been shown in recent research, radiomics can be used to improve the prediction of malignancy and risk stratification. As an illustration, a previous study [17] created a radiomic model that was based on retrospective data of 875 nodules (benign and malignant). Radiomic features were derived from CT images. Following feature selection *via* the Least Absolute Shrinkage and Selection Operator (LASSO), the most informative features were incorporated into a logistic regression-based nomogram. The model had an AUC of 0.836 in the training group and 0.809 in the validation group. Although these encouraging outcomes have been achieved, clinical translation is limited by differences in image acquisition protocols, instability of features, and type I errors.

Another model, which was proposed previously [18], is based on eight imaging characteristics, including shape, surface texture, and spatial distribution, without considering usual size parameters. It was tested using 726 nodules in the NLST dataset and a different cohort of 170 incidentally detected nodules (78 benign, 92 malignant), and showed good performance (AUCs of 0.94 and 0.90, respectively).

It is based on this development that a previous study [19] examined 16,797 nodules (513 malignant) from 6,060 participants in four international screening trials. They filtered 2,060 radiomic features to yield 642 radiomic and 9 epidemiological features to be used in the model. The resulting model performed better than the established PanCan model (AUC = 0.87), with an overall AUC of 0.93 for solid nodules and 0.91 for subsolid nodules.

Future studies are exploring the integration of deep learning with radiomics in indeterminate ground glass nodules (GGNs). The study by Hu *et al*. [20] retrospectively studied 513 pathologically confirmed GGNs (100 benign, 413 stage I adenocarcinomas): the segmentation step was conducted using the hybrid framework of 3D U-Net with convolutional and residual architecture, and the classification step was performed using the transfer learning-based deep neural networks (DNNs). The model achieved an AUC of 0.73 ± 0.06. Even though the diagnostic performance is moderate, the research is the first to implement deep learning and radiomics in the evaluation of GGN.

### 1.5 Applications of Radiomics in Noninvasive Risk Stratification of Malignant Pulmonary Nodules (Classification of Lung Cancer Pathological Subtypes)

Patient prognosis is closely related to the histopathological type and invasiveness of lung cancer. It is essential to preoperatively identify the invasiveness and pathological type of malignant nodules to enhance clinical management and patient outcomes. The 2011 multidisciplinary classification system of lung adenocarcinoma developed by the International Association for the Study of Lung Cancer, the American Thoracic Society, and the European Respiratory Society suggests that lung adenocarcinomas can be classified into three groups based on invasiveness: adenocarcinoma in situ (AIS), minimally invasive adenocarcinoma (MIA, with invasive foci ≤5 mm), and invasive adenocarcinoma (IAC). For MIA, sublobar resection combined with limited lymph node dissection is recommended. This results in a 5-year survival rate of 100%. In the case of IAC, lobectomy and standard lymph node dissection are the preferred procedures, which have a 5-year survival rate of 74.6% [21]. Therefore, the correct preoperative distinction between MIA and IAC is of high clinical value.

Risk stratification by current preoperative biopsy methods (fine or core) is, however, not reliable because of sampling constraints such as small tissue volume and inappropriate sampling [22]. This has made postoperative surgical pathology continue to be the main source of histological classification. A virtual biopsy tool or noninvasive risk stratification model of lung adenocarcinoma is therefore urgently needed clinically, and radiomics-based research is currently underway to fulfill this need.

A number of investigations have already reported promising outcomes [23]. Retrospectively analyzed clinical, pathological, and imaging data from 146 mixed ground-glass nodules (mGGNs) of 141 patients. By performing image segmentation, feature extraction, and feature selection, they found that nodule mass and volume were statistically significant independent differentiators. According to the ROC curve, the optimal cut-off mass to differentiate pre/minimally invasive adenocarcinoma from invasive adenocarcinoma was 254.87 mg, with a sensitivity of 84.52%, a specificity of 88.71%, and an accuracy of 86.30%. The majority of existing literature has employed single time-point CT scans in the prediction of the pathological subtype of ground-glass nodules. Nevertheless, a previous study offered a new longitudinal design based on follow-up CT scans. They gathered 383 serial CT images of 148 patients and designated cases based on postoperative pathology [24]. Vectors representing temporal variations in the extracted features were then used to build a subspace k-nearest neighbor (K-NN) classifier. The model achieved an AUC of 0.725, which was superior to the normal clinical benchmark of 0.675.

Besides CT, several studies have investigated PET-CT-based radiomics as a noninvasive risk stratification tool for pulmonary malignant nodules. A study earlier analyzed pretreatment PET scans of 358 stage I–III non-small cell lung cancer (NSCLC) patients across seven centers in the period from October 2008 through December 2013, with 249 stage I–III patients succumbing to the disease during follow-up [25]. They determined 665 three-dimensional radiomic features in seven grayscale levels in each primary tumor. The most predictive feature vector discovered (FVX) was found to be independent of conventional prognostic factors such as tumor stage, tumor size, and PET-CT scanner model, with high applicability in multicenter cohort studies. This result emphasizes the possibility of pretreatment PET-CT radiomics to risk-stratify patients with NSCLC noninvasively.

Since the field of radiomics is evolving rapidly, virtual biopsy tools and noninvasive stratification models will hopefully be applicable in the near future to help personalize treatment for lung adenocarcinoma patients.

### 1.6 The Role of Radiomics in Identifying Lung Cancer-Associated Genetic Mutations (Radiogenomics)

The most prevalent type of non-small cell lung cancer (NSCLC), which is usually linked to genetic mutations such as epidermal growth factor receptor (EGFR) and Kirsten rat sarcoma (KRAS), as well as aberrant protein expression of programmed death-ligand 1 (PD-L1), is lung adenocarcinoma. The use of therapies targeting these molecular features, including tyrosine kinase inhibitors (TKIs), ALK inhibitors, and immune checkpoint inhibitors (ICIs), has become a routine aspect of personalized therapy for EGFR-mutant and PD-L1-positive lung adenocarcinoma [26, 27]. According to the guidelines of the Chinese Society of Clinical Oncology (CSCO), EGFR-TKIs are recommended as first-line therapy since they have been shown to provide better survival rates than chemotherapy in EGFR-mutated metastatic adenocarcinoma [28]. Immunotherapy is hence a novel pillar in the management of lung cancer in its advanced stage.

Up to this point, invasive biopsy is the most common form of EGFR mutation testing and subtype typing. Noninvasive alternatives, however, are provided by radiomics, which predicts both pathological subtypes as well as genetic mutations based on imaging data. One such tool is the Computer Aided Nodule Assessment and Risk Yield (CANARY) system created by [18, 29]. It is a virtual biopsy tool that is developed based on nine features of CT-based images to predict pathological subtypes of lung adenocarcinoma. CANARY demonstrated EGFR mutation prediction potential (AUC = 0.77), and this result improved to 0.87 when clinical variables such as smoking status were supplemented with imaging features, but not with KRAS mutations [30].

A study mentioned earlier analyzed CT images of 351 patients (262 for training, 89 for validation) and extracted 636 radiomic features. Their 13-feature model achieved AUCs of 0.51–0.76 for KRAS, 0.51–0.68 for EGFR, and 0.51–0.65 for TP53 mutations [31]. Similarly, a previous study established that radiomic features performed better as compared to volumetric features (*e.g*., tumor size) in predicting EGFR mutations and TKI response [32]. Another study constructed a 13-feature radiomic model, which predicts the presence of EGFR mutation (AUC = 0.7413), and discovered that a smaller skew (≤ 0.882) and a smaller 10th-percentile value (≤ 21.132) were related to a higher partial response rate (P = 0.01) [33].

The high-end AI applications have also supported radiogenomic analysis. A previous study created a multitask AI system, which was a combination of deep learning (DL), radiomics (RA), and joint (JO) modules to predict the status of EGFR and PD-L1 using CT scans. The RA module, trained on data from 4,404 patients with NSCLC (3,816 used for testing the model), achieved AUCs of 0.805 (EGFR) and 0.795 (PD-L1), whereas the JO module achieved 0.928 and 0.905, respectively [34]. Likewise, a previous study assessed radiomics-based (MRadiomics) and multi-scale residual convolutional neural networks (MCNNs) models in 1,010 patients (510 EGFR-mutant, 500 wild-type). The MCNNs model (AUC = 0.810) was superior to MRadiomics (AUC = 0.740, P = 0.0225) [35]. However, combining the two models did not yield further improvement (AUC = 0.811), suggesting potential model redundancy or context-specific limitations. Besides CT, Zhang, *et al*. also applied radiomic analysis to PET-CT scans, which was found to be effective in identifying mutations linked to lung cancer and thereby increasing noninvasive genetic profiling [36].

The developments suggest that radiogenomics may be employed as an extension of, or even as an alternative to, invasive biopsies in the future. Nevertheless, certain issues related to feature reproducibility, model robustness, and generalizability across institutions still exist and require further investigation.

### 1.7 The Function of Radiomics in Predicting Treatment Response and Recurrence in Lung Cancer Patients

One of the most pertinent problems in lung cancer management is predicting treatment response and estimating the likelihood of recurrence or distant metastasis. In current clinical practice, treatment efficacy is primarily assessed using the Response Evaluation Criteria in Solid Tumors (RECIST 1.1) [37]. However, RECIST is associated with significant limitations in terms of continuous and accurate monitoring of treatment outcomes. These gaps are being addressed by radiomics-based investigations that assess post-operative, post-radiotherapy, post-chemotherapy, post-targeted therapy, or post-immunotherapy changes and predict recurrence and metastasis.

In 2023, Wang, *et al*. conducted a systematic review and meta-analysis of CT-based radiomics for predicting outcomes in EGFR TKI-treated NSCLC [38]. They summarized the existing global evidence up to April 2023 and found that pooled radiomic features were strong predictors of progression-free survival (PFS), with a hazard ratio (HR) of 2.80 (95% confidence interval: 1.87–4.19). The pooled Harrell’s concordance index (C-index) and AUC were 0.71 and 0.73, respectively, affirming the strong prognostic power of radiomics in EGFR TKI therapy.

The CANARY model, which was previously developed as a virtual biopsy tool for noninvasive risk stratification [29, 39], was also demonstrated to classify post-treatment outcomes as good, moderate, or poor, thereby being useful in predicting surgical resection outcomes in lung cancer. A previous study compared pre- and post-treatment non-contrast CT scans of 172 patients who received EGFR TKI therapy for lung adenocarcinoma (LUAD) [40]. The authors selected 10 important features using intratumoral and peritumoral radiomic features and the Boruta selection algorithm. Their radiomic model achieved AUCs of 0.78 (95% CI: 0.72–0.84) in the training cohort and 0.72 (95% CI: 0.60–0.84) in the validation cohort, outperforming clinical feature models (AUCs of 0.55 and 0.54, *P* < 0.001).

A previous study evaluated radiomic features extracted from multiphasic CT (non-contrast, arterial, and venous phases) to predict EGFR mutation status and TKI sensitivity in 253 patients with advanced LUAD [41]. Among the non-contrast, arterial, and venous phase features obtained using LASSO regression and 10-fold cross-validation, the venous phase features showed better performance in predicting EGFR mutation status (AUC = 0.808) and TKI sensitivity (AUC = 0.895), implying that venous-phase radiomics is an effective biomarker for predicting TKI responsiveness.

In a similar investigation, Dou, *et al*. showed that malignant cells may survive in seemingly normal tissue near the primary lesion and thus facilitate metastasis [42]. They found that a 3-mm peritumoral radiomic architecture was associated with an increased risk of distant metastasis. This model (C-index = 0.64) outperformed multivariate clinical models (0.53), visible tumor radiomics models (0.59), and tumor external tissue models (0.55), demonstrating the clinical importance of peritumoral radiomics.

### 1.8 Challenges and Future Directions of Radiomics in Pulmonary Nodule Management

Even though there has been significant growth in the clinical use of radiomics for the diagnosis and treatment of pulmonary nodules, a number of issues still remain. As mentioned above, radiomics models tend to perform well only when applied to images obtained using specific protocols within single imaging centers. Therefore, the lack of standardization in imaging acquisition processes is a major challenge to the clinical implementation of radiomics. Independent training and validation of models on heterogeneous, multi-institutional datasets to achieve broader generalizability represent a potential direction for future research.

Interpretability, reproducibility, and performance stability also continue to pose challenges in model development. Such algorithms are less acceptable to clinicians because they may not fully understand the principles underlying complex machine learning algorithms, such as random survival forests. In addition, limited data sharing among research teams restricts the possibility of model replication and external validation. Current radiomics workflows, from image acquisition to feature extraction, remain labor-intensive because of their reliance on manual segmentation, which introduces subjectivity and is time-consuming.

Despite the fact that deep learning-based radiomics (DLR) allows automated feature extraction using latent representations, its lack of interpretability and reproducibility makes it less likely to be adopted in clinical practice [43]. Therefore, there is a need to enhance the integration of deep learning and traditional radiomics in future research.

Overall, radiomics has enormous potential for the diagnosis, prognosis, and treatment of pulmonary nodules. However, addressing challenges related to data standardization, model transparency, reproducibility, and clinical interpretability will be essential for its incorporation into routine clinical practice.

## CONCLUSION

Radiomics has become a potent, non-invasive tool with significant potential for the accurate diagnosis, characterization, and treatment of pulmonary nodules and lung cancer. Radiomics enables accurate lesion segmentation, automatic feature extraction, and objective evaluation of morphological, texture-, and intensity-based characteristics from conventional medical images by transforming them into high-dimensional, quantifiable data. Advances in imaging technology, artificial intelligence, and deep learning are driving the development of more robust and generalizable models, despite existing challenges related to standardization, reproducibility, and clinical integration.

## Figures and Tables

**Fig. (1):**
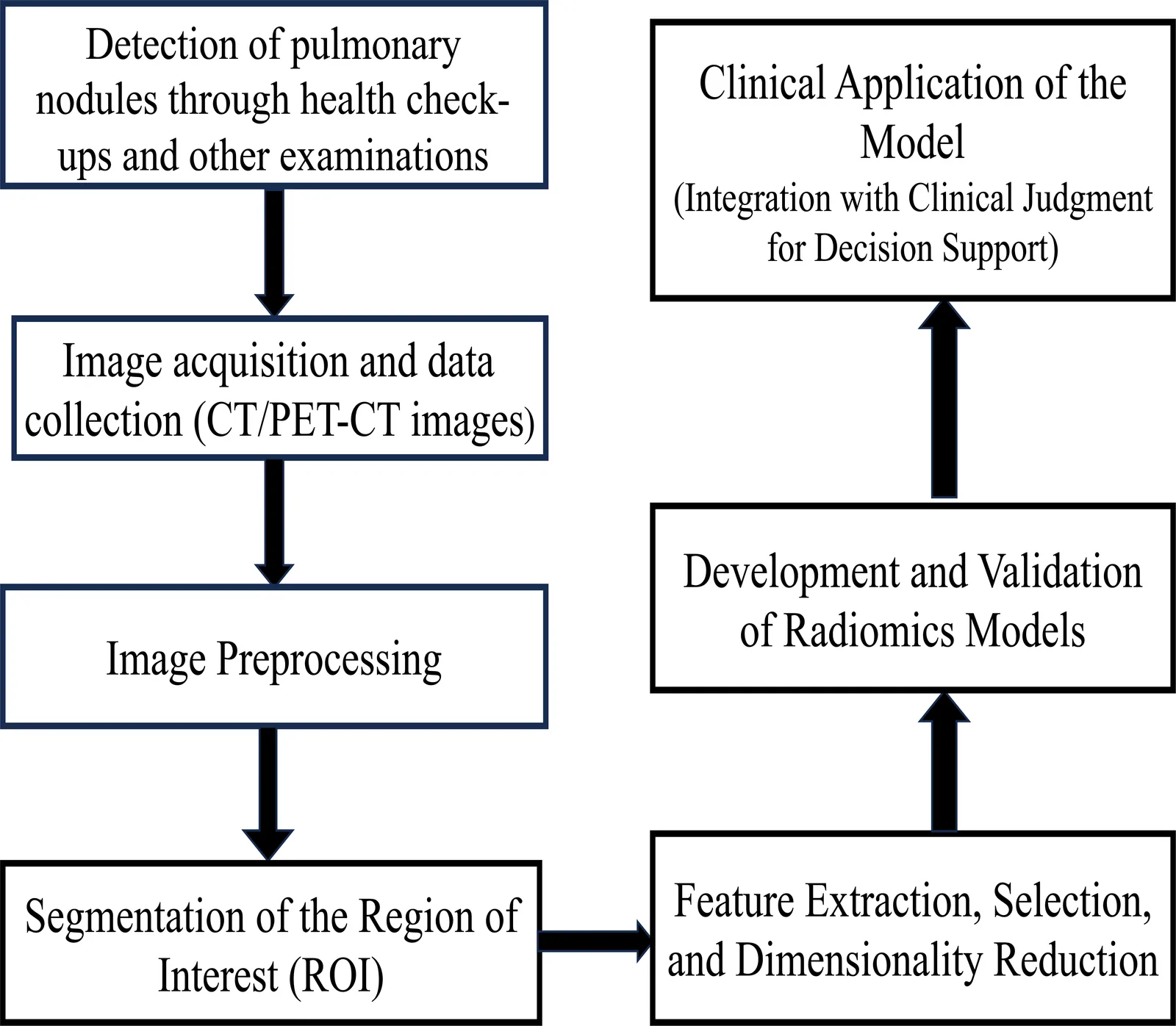
Workflow of pulmonary radiomics.

**Fig. (2):**
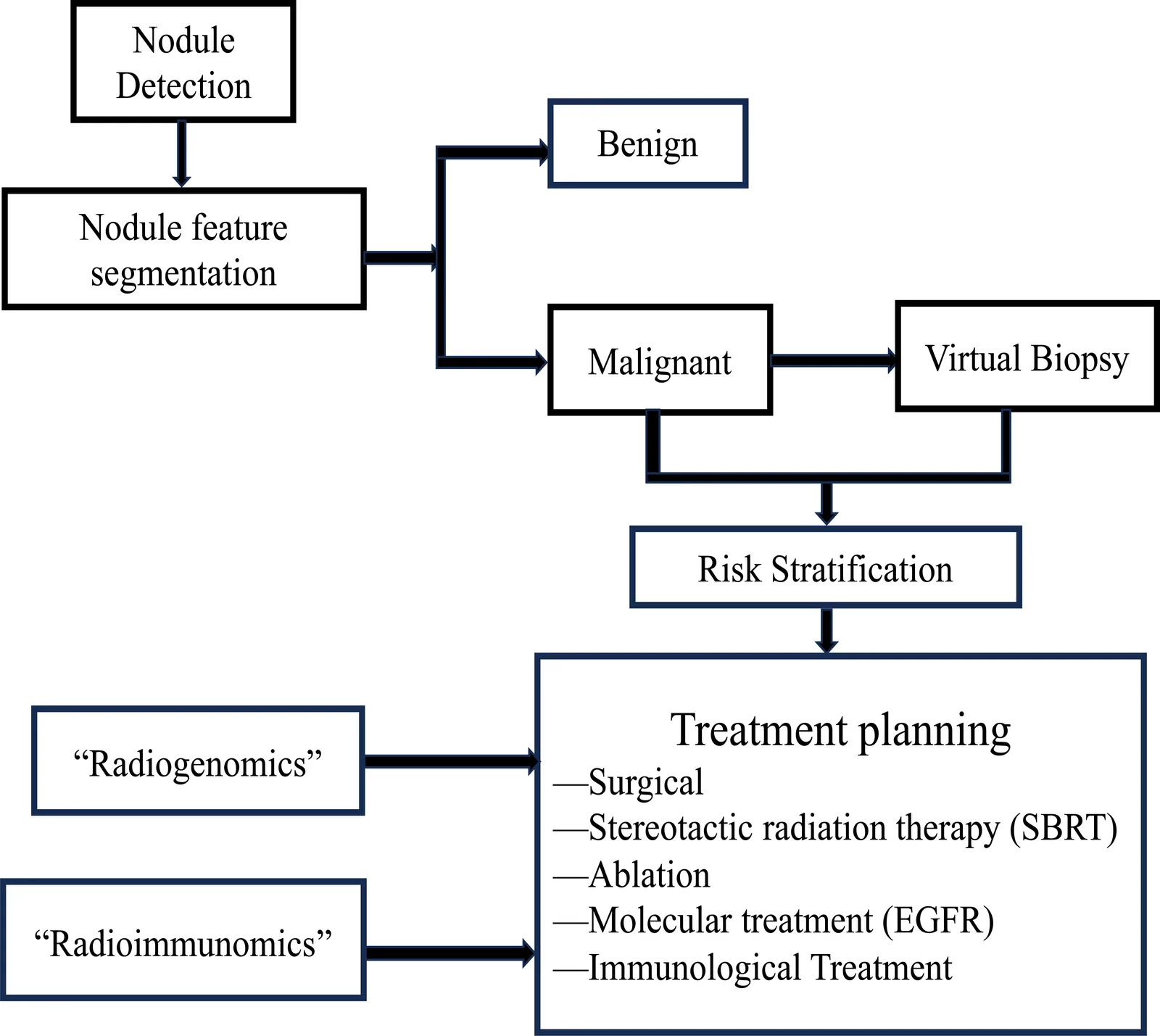
Radiomics in pulmonary nodule and lung cancer management.

## LIST OF ABBREVIATIONS

AIS: Adenocarcinoma in Situ
AI: Artificial Intelligence
ALK: Anaplastic Lymphoma Kinase
AUC: Area Under the Curve
CANARY: Computer-Aided Nodule Assessment and Risk Yield
C-index: Concordance Index
CNN: Convolutional Neural Network
CSCO: Chinese Society of Clinical Oncology
CT: Computed Tomography
DLR: Deep Learning-based Radiomics
DNN: Deep Neural Network
EGFR: Epidermal Growth Factor Receptor
GGN: Ground-Glass Nodule
HRCT: High-Resolution Computed Tomography
IAC: Invasive Adenocarcinoma
ICI: Immune Checkpoint Inhibitor
KRAS: Kirsten Rat Sarcoma
K-NN: K-Nearest Neighbor
LASSO: Least Absolute Shrinkage and Selection Operator
LUAD: Lung Adenocarcinoma
MCNNs: Multi-scale Residual Convolutional Neural Networks
MIA: Minimally Invasive Adenocarcinoma
mGGN: Mixed Ground-Glass Nodule
MRI: Magnetic Resonance Imaging
NSCLC: Non-Small Cell Lung Cancer
PD-L1: Programmed Death-Ligand 1
PET-CT: Positron Emission Tomography–Computed Tomography
PFS: Progression-Free Survival
RECIST: Response Evaluation Criteria in Solid Tumors
ROI: Region of Interest
TKI: Tyrosine Kinase Inhibitor
TMB: Tumor Mutational Burden
